# Nutrient Addition Has a Stronger Effect Than Intraspecific Genetic Diversity on Critical Ecological Responses in a Salt Marsh Foundation Species

**DOI:** 10.1002/ece3.72908

**Published:** 2026-01-12

**Authors:** Jewel Tomasula, Billie Maguire, Tyler M. Rippel, Shannon M. Murphy, Matthew B. Hamilton, Gina M. Wimp

**Affiliations:** ^1^ Department of Biology Georgetown University Washington DC USA; ^2^ Department of Biology University of Massachusetts Boston Boston Massachusetts USA; ^3^ Department of Biology University of Denver Denver Colorado USA

**Keywords:** genetic diversity, genotype by environment interaction, genotypic identity, nutrient addition, plant population and community dynamics, salt marsh, *Spartina alterniflora*

## Abstract

Intraspecific genetic diversity in wild populations is declining as global change intensifies. Genetic variation within populations of foundation plant species may influence ecological responses to environmental stressors, but remains poorly understood. Here, we examined how intraspecific genetic diversity in salt marsh cordgrass ( *Spartina alterniflora* , hereafter *Spartina*) affects responses to nutrient addition. Salt marshes are often dominated by *Spartina*, a partially clonal foundation species that is critical to the structure and ecosystem function of salt marsh habitats. We conducted a two‐way factorial greenhouse experiment to examine plant responses to two levels of intraspecific genetic diversity and two levels of nutrient addition. We genotyped plants and estimated genetic distance and clonal identities. We measured aboveground biomass, belowground biomass, tiller production, and percent nitrogen content of leaf tissue. Nutrient addition had a strong main effect on combined plant responses, but we did not find an impact of intraspecific genetic diversity or the interaction between intraspecific genetic diversity and nutrient addition on the combined plant responses. When we examined plant responses individually, we found that nutrient addition decreased belowground biomass and increased leaf tissue nitrogen content. Nutrient addition interacted with intraspecific genetic diversity to affect tiller production, whereby nutrient addition increased tillers in low genetic diversity pots, but decreased tillers in high genetic diversity pots. Unexpectedly, neither nutrient addition nor intraspecific genetic variation affected aboveground biomass, which may be driven by divergent responses among different plant clones. For example, of the four most common clones (Multilocus Lineages), nutrient addition increased aboveground biomass in the low diversity treatment for two clones, and in the high diversity treatment for another clone. Unlike the well‐established relationship between plant interspecific diversity and ecosystem function, intraspecific genetic diversity of a foundation species did not affect plant responses at a local scale to nutrient addition.

## Graphical abstract

Intraspecific genetic diversity of a foundation species did not affect plant traits at a local scale, or resilience to nutrient inputs. However, variation among clones in response to nutrient enrichment and intraspecific genetic diversity supports the idea that genetic variation may still be important at larger spatial scales.

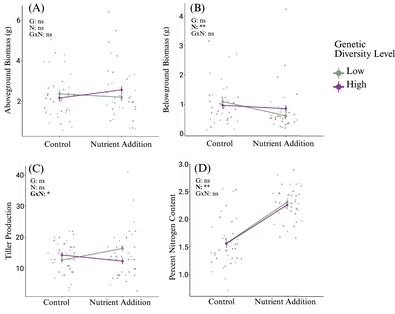

## 1 Introduction

Genetic diversity appears to be declining as part of the current global biodiversity crisis (Exposito‐Alonso et al. 2022). Losing genetic diversity within populations of foundation plant species is problematic because it can have broader ecological consequences, such as affecting dependent animal community structure and ecosystem function; thus, losing genetic diversity may have effects beyond the population‐level (Wimp et al. 2004; Reusch et al. 2005; Whitham et al. 2006; Hughes et al. 2008; Raffard et al. 2018). Plant genetic diversity in of itself has been a useful predictor of ecological responses in many systems because plant phenotypes are often polygenic with multiple genes underlying traits that impact ecological processes (Bailey et al. 2009; Abbott et al. 2017). In addition to plant genetic diversity having an impact on community and ecosystem processes, such diversity may directly affect the focal plant population. Intraspecific genetic diversity may mediate how plants respond to environmental factors, and thus how entire ecosystems are impacted by rapidly changing environmental conditions and ubiquitous anthropogenic impacts of the Anthropocene (Reusch et al. 2005; Raffard et al. 2018). For example, following a rise in sea surface temperatures, eelgrass shoot density and biomass were greater in plots with higher genotypic diversity (Reusch et al. 2005). The interaction between intraspecific genetic diversity and environmental change is especially relevant in ecosystems with naturally low plant species richness and where a dominant species reproduces vegetatively (i.e., clonally; Reusch and Hughes 2006; Barrett 2015). These monodominant ecosystems occur globally, including intertidal wetlands, non‐tropical forests, and grasslands, and many are particularly vulnerable to anthropogenic impacts (Reusch and Hughes 2006, Barrett 2015).

One human activity that undermines ecological integrity is nutrient enrichment (Tilman 1999; Howarth et al. 2002; Malone and Newton 2020). Chronic influxes of nutrients have been shown to impact biodiversity in a variety of ecosystems, altering plant species composition and food web structure (Tilman 1987; Inouye and Tilman 1995; Bertness et al. 2002). Changes in nutrient availability can have strong direct impacts on plant performance, as well as indirect impacts through biotic interactions, like competition and complementarity. Generally, nutrient addition increases plant biomass, but increased plant biomass can also be driven by niche complementarity or by “sampling effects” (Tilman 2004; Hooper et al. 2005; Tilman et al. 2014). Niche complementarity happens when higher species diversity is correlated with higher diversity in resource use among the different species; this minimizes competition and results in greater productivity in diverse communities relative to monocultures of the most productive species (Tilman et al. 2001). Alternatively, higher productivity can also be found in diverse communities due to the sampling effect, whereby increased species diversity also increases the likelihood that the most productive species is present in a community (Hooper et al. 2005). These mechanisms are most often tested among species, not within species, but similar mechanisms can operate at the intraspecific level. For example, plant productivity increases in polycultures that include a stress‐tolerant genotype relative to monocultures (Zerebecki and Hughes 2023). At the intraspecific level, studies have largely been conducted under ambient conditions without manipulating an environmental factor (Hughes et al. 2008; but see Parker et al. 2010, Limbu and Avolio 2022, Zerebecki and Hughes 2023), or do not measure genetic diversity directly, but use genotypic diversity (i.e., clonal diversity) as a proxy. Measurements of genetic diversity are important because high clonal diversity in one population may represent a relatively low amount of genetic diversity compared to another population, making synthesis and among‐population and among‐system comparisons difficult. Thus, there is a need to test how intraspecific genetic diversity can affect plant responses to the environmental factor of nutrient addition. For instance, do low diversity populations respond differently to nutrient inputs than high diversity populations by virtue of heterogeneous nutrient responses by different genotypes? Answers to this question will provide insight into how ecosystems respond to simultaneous genetic diversity loss and ongoing anthropogenic nutrient addition.

To investigate how intraspecific genetic diversity, nutrient addition, and their potential interaction influence plant responses, we conducted a two‐way factorial greenhouse experiment with the salt marsh foundation species *Spartina alterniflora* (hereafter *Spartina*). A partially clonal grass species, *Spartina* forms monospecific expanses that naturally cover the low salt marsh habitats. At the interface of terrestrial and marine systems, salt marshes provide numerous societal benefits and have a distinct ecological importance (Gedan et al. 2009; Rippel et al. 2021). Salt marshes are also in decline globally as they are impacted by a myriad of anthropogenic stressors (Gedan et al. 2009, Rippel et al. 2021). Nutrient enrichment is one of the most urgent stressors on estuarine ecosystems and has been linked to salt marsh collapse (Deegan et al. 2012). Previous work has shown that *Spartina* readily responds to nutrient inputs with changes in biomass and tissue chemistry (Wimp et al. 2010; Murphy et al. 2012; Deegan et al. 2012; Johnson et al. 2016). While short‐term increases in nitrogen (pulses) can have a positive impact on plants, repeated nitrogen inputs in the form of a nutrient press can lead to plant die‐back due to nutrient toxicity, decreases in belowground biomass, and increased attack by insect herbivores (Deegan et al. 2012; Tomasula et al. 2023). Because *Spartina* exclusively dominates the habitat and its root structures hold sediment in place, its biomass determines productivity and structural integrity of the salt marsh ecosystem. Further, vegetative growth and tiller production may respond to nutrient inputs and/or vary among genotypes, with the potential to alter reproduction by clonal growth versus sexual reproduction and thereby the spatial distribution of intraspecific genetic variation (Barrett 2015). If intraspecific patterns follow interspecific patterns, nutrients and diversity would additively affect plant production in the short term (with genetic diversity having a larger effect over time, Tilman et al. 2012). Because there is foundational knowledge of *Spartina* responses to nutrient inputs (e.g., Gratton and Denno 2003; Murphy et al. 2012; Wimp et al. 2019; Rippel et al. 2021), it is an ideal system to examine how the level of intraspecific genetic diversity may alter how key plant traits respond to nutrient addition.

In this study, we ask: (1) How do *Spartina* aboveground biomass, belowground biomass, tiller production, and leaf percent nitrogen content respond to nutrient addition and intraspecific genetic diversity? (2) How do distinct *Spartina* genets (i.e., genetically distinct groups originating from sexual reproduction) respond to nutrient addition and intraspecific genetic diversity? We anticipated finding interactive effects of nutrient addition and intraspecific genetic diversity on aboveground and belowground biomass, tiller production, and percent nitrogen content of leaf tissue.

## 2 Materials and Methods

### 2.1 Study Species

*Spartina alterniflora* (also known by *Sporobolus alterniflorus*; hereafter *Spartina*) dominates salt marsh ecosystems along the Atlantic coast of the Americas at elevations below mean high tide, forming monospecific habitats (Bertness 1991; Denno et al. 2002). As a foundation species (*sensu* Ellison 2019), *Spartina* is a structural and nutritional resource underpinning salt marsh food webs. *Spartina* reproduces sexually by inflorescences and seeds as well as vegetatively by rhizomes and tillers. Previous research demonstrated that vegetative offspring in the study population are highly aggregated and unlikely to spread more than 2 km (Tomasula 2023).

**FIGURE 1:**
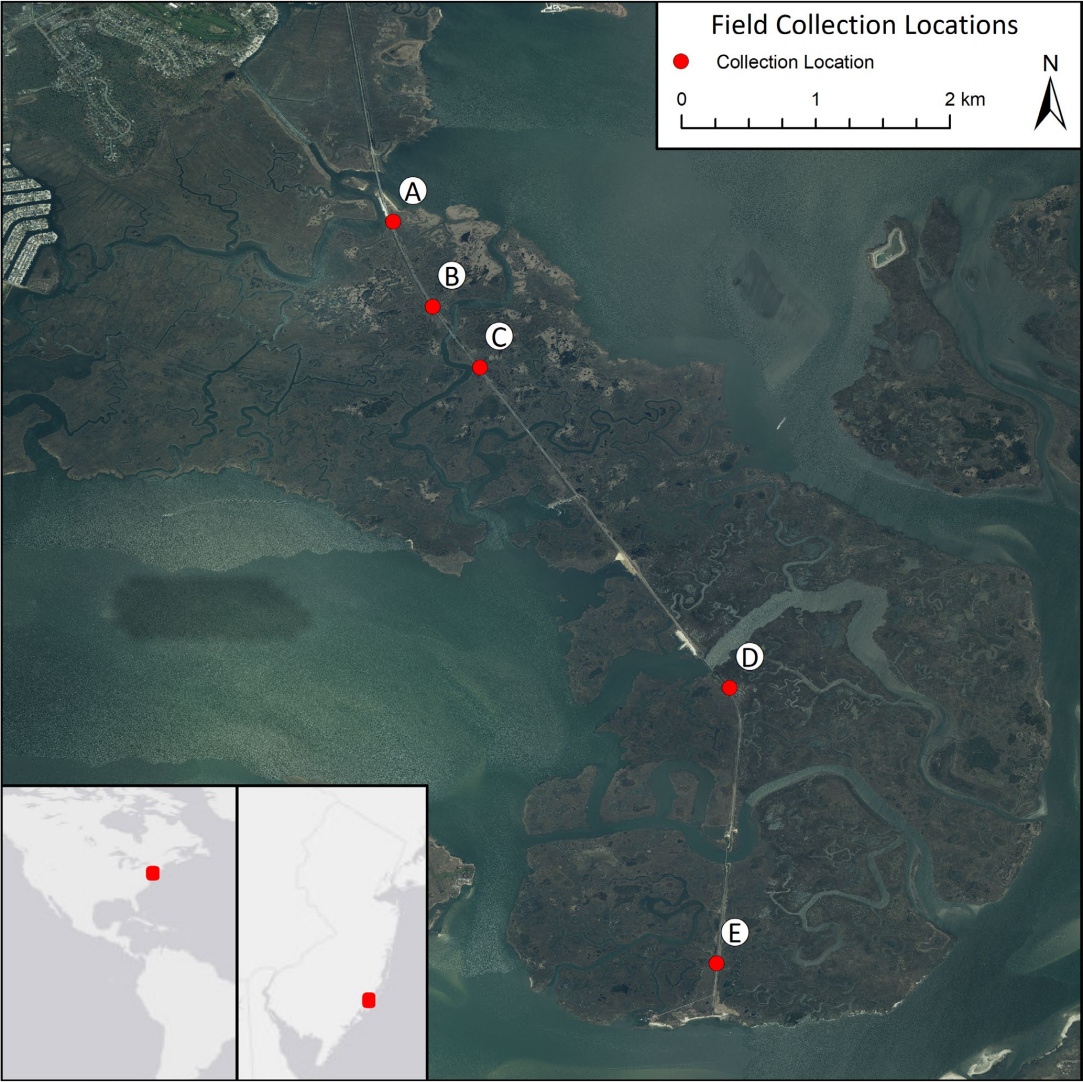
Collection locations of *Spartina alterniflora* ramets in the Jacques Cousteau National Estuarine Research Reserve near Tuckerton, New Jersey, United States.

For our experiment, we collected approximately 500 *Spartina* plants from five locations (A, B, C, D, and E) in the Great Bay Boulevard Wildlife Management Area in Ocean County, New Jersey USA in early July 2020 (Figure 1). The estuary has been well‐protected for decades, exhibits one of the lowest nitrogen densities found in plants from Virginia to Maine, does not experience salt marsh collapse, and is one of the least impacted salt marshes on the United States eastern seaboard (Kennish and O'Donnell 2002; Wimp et al. 2019). However, nutrient uptake (as measured by nitrogen density or the live biomass per m^2^ × percent nitrogen found in the plants) differed among sites, with higher nitrogen density at the A (average of 5.3) collection site, but no difference in nitrogen density among sites B–E (average of 3.9, 3.5, 3.2, and 3.0, respectively). Our aim was to obtain a range of genetically similar plants to genetically distant plants. Thus, our collection locations ranged from approximately 0.8 km to 6 km apart, and at each location we collected plants directly adjacent to each other to the extent possible and at most 0.1 km distance. We constructed this sample scheme with the expectation that genetically distant plants would be found among more distant locations, while identical and more genetically similar plants would be found within locations, as predicted by the clonal life history of the species and by isolation by distance (Wright 1943; Vekemans and Hardy 2004). The maximum distance between locations (6 km) was constrained by the size of the peninsula and urban encroachment (Figure 1); for the questions we were interested in testing, we needed to sample from the same breeding population so that we were studying genetic diversity at a scale that was ecologically relevant for our study species. Moreover, we intended the sampled plants to reflect the range of genetic diversity within the natural population, which is why we collected all plants across the Great Bay Boulevard Wildlife Management Area instead of collecting across a region.

### 2.2 Experimental Setup

In early July 2020, the day after collection, we set up a two‐way factorial greenhouse experiment crossing intraspecific genetic diversity (low, high) with nutrient addition (control, nutrient addition). Due to institutional restrictions on laboratory access during the onset of the COVID‐19 pandemic in the USA, we were unable to genotype plants before setting up the experiment, but did so afterwards. To set up our experimental pots before genetic analyses could be done (see *Characterization of intraspecific genetic diversity* below), we used collection location as a proxy for genetic diversity, a method widely applied in research as well as conservation and restoration practice (Williams et al. 2014; Gaynor et al. 2019).

We set up experimental pots with 5 plants in each pot, using 15.2 cm diameter garden pots that were filled with a mixture of one‐part sand and four‐part Miracle‐Gro all‐purpose garden soil. To vary genetic diversity, we set up pots with plants: (1) from a single collection location (e.g., all five plants from location A, all five plants from location B, etc. for a total of 5 locations), (2) from 3 collection locations (i.e., A–C–E with 2 plants from A, 1 plant from C, 2 plants from E; B–C–D with 2 plants from B, 1 plant from C, and 2 plants from D), and (3) with one plant from each of the five locations (A–B–C–D–E). Thus, we had 8 different treatments (5 single source, 2 three source, and 1 five source) that were replicated 10 times each for a total of 80 pots. Each plant within each pot was individually labeled and we measured the initial stem height (cm) and initial number of tillers. We took leaf snips of each plant for genotyping and stored them at −20°C until genetic analyses could be performed. We haphazardly assigned 5 pots from each treatment to receive nutrient addition, and the remaining 5 pots were assigned to control. In each of the nutrient addition pots, we applied a total of 2.2 g urea and 0.88 g triple phosphate as granular, slow‐release fertilizer; nutrient uptake occurs within a few days of application (unpublished data). We applied half the fertilizer 1 week after establishing plants in the pots and the other half 2 weeks after. We chose the nutrient addition amount to correspond to 54.4 g N/m^2^ applied in previous experiments (Tomasula et al. 2023) and to correspond to highly enriched sites in natural settings that border urban development (Wimp et al. 2019). For the duration of the experiment, we haphazardly shuffled pot locations within the greenhouse on a twice weekly basis for a completely randomized design.

### 2.3 Quantifying Plant Responses

At the end of the experiment (8 weeks later, chosen for the timing of peak biomass in *S. alterniflora* at the end of the growing season, Materne et al. 2022), we removed plants from pots, carefully separating their belowground structures from each other and discarding soil. Because we marked the original stem and carefully dissected all individuals that were attached to the original stem via rhizomes, we were able to count the number of tillers produced by each individual stem. We counted the number of tillers for each individual plant, subtracting the initial tiller count to estimate tiller production. We rinsed plants, separated aboveground and belowground biomass and placed samples into separate, labeled paper bags. We stored the samples at −20°C. Once we regained access to the laboratory, we dried samples in a drying oven at 60°C for 3 days and then measured the aboveground biomass (g) and belowground biomass (g) for each sample. We haphazardly subsampled the dried leaf tissue, ground them in a Retsch MM 400 Model mixer mill (Retsch GmbH, Haan, Germany), weighed them using a Mettler‐Toledo XP6 microbalance (Mettler‐Toledo, Columbus, OH), and rolled them into tin capsules (Elementar Americas). We sent these prepared samples to the Cornell Stable Isotope Laboratory to be analyzed for %N analysis using an elemental analyzer‐stable isotope ratio mass spectrometer system (Thermo Delta V Advantage IRMS and Carlo Erba NC2500 EA systems).

### 2.4 Characterization of Intraspecific Genetic Diversity

To determine genotypes of the plants used in experimental treatments, total genomic DNA was extracted from approximately 2 mg of leaf tissue using a Chelex‐based protocol modified from Walsh et al. (1991) for *Spartina* spp. We employed 10 highly polymorphic microsatellite genetic marker loci after screening over two dozen *Spartina* microsatellite loci described by Blum et al. (2004) and Sloop et al. (2005) as detailed in Tomasula et al. (2023). Two multiplex sets were used (Spar05, Spar19, Spar34, Spar07, and Spar02, Spar09, Spar16, Spar20, Spar23, Spar26) with fluorescent dye labeled primers and amplicon sizes to distinguish loci. Multiplex sets were amplified with Qiagen Type‐it microsatellite PCR reagents. PCR products from both multiplex amplifications from one sample were combined, mixed with GeneScan 600 LIZ size standard in formamide, and fragments separated by capillary electrophoresis with POP 7 polymer on an ABI 3500 Genetic Analyzer. Microsatellite alleles were scored by binning fragments using the microsatellite plug‐in for Geneious Prime (version 2022.2.2) with a 3rd order least‐squares sizing curve. Multilocus genotypes were determined for a total of 79 pots (395 *Spartina* individuals) after elimination of one pot with insufficient DNA for PCR amplification.

To distinguish unique genets by microsatellite multilocus genotypes (MLG) and thereby identify multilocus lineages, or MLL (*sensu* Arnaud‐Haond et al. 2007), we used the poppr R package (Kamvar et al. 2015) and Genodive v3.06 (Meirmans 2020) to estimate pairwise Bruvo genetic distances (Bruvo et al. 2004) with a stepwise mutation model. We estimated a threshold genetic distance, ignoring any missing genotype data, below which MLG were considered to belong to the same MLL. This thresholding mitigates the impact of scoring errors, as well as any very recent mutations (Arnaud‐Haond et al. 2007). To evaluate the power of the MLG to distinguish MLLs, or the chance of observing MLG identical by chance and incorrectly assigning them to the same MLL, we estimated the probability of exclusion, (1—*p*_sex_(*F*))^*N*^ where *N* was the number of samples. The RClone R package (Bailleul et al. 2016) was used to estimate average expected genotype frequencies with sexual reproduction, *p*_sex_(*F*), using the round‐robin allele frequencies method and the fixation index option. Genetic differentiation between pairs of sampling locations was estimated with *F*_*ST*_ and *R*_*ST*_ using only the MLLs in Genodive v3.06. A neighbor joining tree was estimated using the Bruvo's genetic distances for MLLs.

For each pot (*n* = 5 plants), we estimated the MLL richness and the mean pairwise genetic distance. The mean pairwise genetic distance was the average of the 10 unordered pairwise genetic distances among MLLs within the pot. We graphed mean pairwise genetic diversity versus genotypic richness to examine the distribution of *Spartina* genetic diversity within pots and inform our low and high intraspecific genetic diversity categories. We categorized pots with mean pairwise genetic distances < 0.50 as “low” and ≥ 0.50 as “high” which defined groups with approximately equal sample sizes. For the “low” and “high” genetic distance groups we estimated the mean number of MLLs per pot, mean of per pot Bruvo's genetic distance, and genetic differentiation between the groups with *F*_*ST*_ and *R*_*ST*_ using only the unique MLLs within each group.

### 2.5 Statistical Analysis

We analyzed the effect of nutrient addition and intraspecific genetic diversity on plant responses (aboveground biomass, belowground biomass, tiller production, and percent nitrogen content of leaf tissue) using MANCOVA. We then examined the impact of nutrient addition and intraspecific genetic diversity on individual response variables using an ANCOVA with initial height as a covariate. While initial heights differed among sampling locations, we did not find initial differences in stem height among treatments due to fertilization (*F*_1,75_ = 0.132, *p* = 0.718), genetic distance category (*F*_1,75_ = 0.226, *p* = 0.636) or the interaction between fertilization and genetic distance category (*F*_1,75_ = 1.341, *p* = 0.251) since all sampling locations were represented across the different treatments. We used pots as replicates (control low diversity treatment: *n* = 17 pots; control high diversity treatment: *n* = 22 pots; nutrient addition low diversity treatments: *n* = 16 pots; and nutrient addition high diversity: *n* = 24 pots). Notably, while we aimed for 20 pots per treatment, our uneven sample sizes were a result of genotyping after treatments had been set‐up due to Covid restrictions; the actual level of diversity within each pot was assessed post hoc and pots were reassigned to diversity treatments after genotyping. Thus, several of our low‐diversity pots ended up being high‐diversity pots. We used the mean initial height of the five plants in the pot as the covariate to account for natural variation in height. We checked response variables for normality with the Shapiro‐Wilks test and homogeneity of variance with the Levene Test. To meet normality assumptions, we log transformed aboveground biomass, belowground biomass, and tiller production. Thus, here we report the more robust Pillai's Trace test statistic, although the *F* and *p* values for Pillai's Trace were identical to those for the Wilk's lambda test statistic.

To examine whether plants of distinct MLLs (clones) respond differently to treatments, we focused on the most abundant MLLs (8, 10, 16 and 17). We used individual plants as replicates and calculated their mean response across pots within treatment groups. MLL replicates per treatment ranged from 3 to 32. We checked for normality and equality of variances, and performed a log transformation when data did not meet assumptions (MLL 8, belowground biomass). We analyzed data using a two‐way ANOVA with intraspecific genetic diversity (low, high) and nutrient addition (control, addition) as factors. Initial height was included as a covariate in the individual two‐way ANOVAs we ran for aboveground biomass, belowground biomass, and nitrogen addition since it was an important factor in our initial MANOVA. We did not include initial height as a covariate in our two‐way ANOVA for tiller production since the original MANOVA did not identify variation in initial height as an important factor.

## 3 Results

### 3.1 Identification of Multilocus Lineages

All loci were highly polymorphic with 1.67% missing single locus genotypes for the 395 samples. The average random match probability for MLG was *p*_sex_(*F*) < 0.0000001, making probability of exclusion with 395 samples greater than 0.999. With a threshold Bruvo's genetic distance of *D* = 0.189 (or an equivalent threshold of 22 in Genodive, Figure S1), we identified 63 MLLs. The observed numbers of MLLs was much less than expected under the null model of sexual random mating (Table S1). *F*_*ST*_ and *R*_*ST*_ estimates for the MLLs between pairs of the five sampling locations (Table S2) and a neighbor joining tree based on genetic distances (Figure S2) all showed that allele frequency and MLL variation was found mostly within rather than among sampling locations and there was no geographic hierarchy of genetic distances. The 35 pots in the low genetic distance category had a mean of 1.9 MLLs and a mean genetic distance of 0.232 while the 44 pots in the high genetic distance category had a mean of 4.1 MLLs and a mean genetic distance of 0.657. The 33 unique MLLs in the low and 54 unique MLLs in the high genetic distance groups did not differ in allele frequencies (*R*_*ST*_ = −0.008, *F*_*ST*_ = −0.005, both *p* > 0.05 by permutation tests).

### 3.2 Treatment Effects on Multivariate and Univariate *Spartina* Responses

Nutrient addition had a strong effect on plant responses (Pillai's Trace = 0.68, *F*_4,71_ = 37.3, *p* < 0.0001). Intraspecific genetic diversity (Pillai's Trace = 0.04, *F*_4,71_ = 0.66, *p* = 0.62) and the interaction of nutrient addition and genetic diversity (Pillai's Trace = 0.06, *F*_4,71_ = 1.21, *p* = 0.31) did not affect plant responses. The covariate, initial stem height, had a strong effect on plant responses.

**FIGURE 2:**
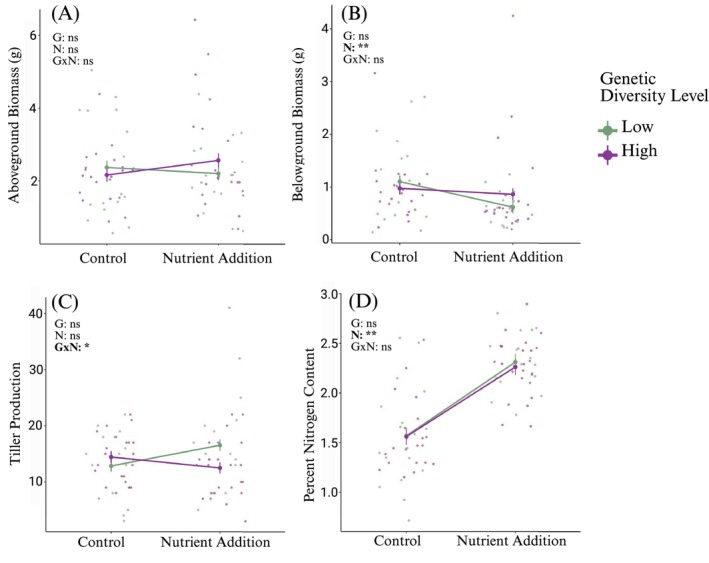
Mean *Spartina alterniflora* aboveground biomass in grams (A), belowground biomass in grams (B), tiller production in number of stems (C), and percent nitrogen content (D) (±SE) to the factorial treatment effects of nutrient addition (N), intraspecific genetic diversity (G), and interaction (G×N). Control low diversity treatment: *N* = 17 pots; control high diversity treatment: *N* = 22 pots; nutrient addition low diversity treatment: *N* = 16 pots; and nutrient addition high diversity treatment: *N* = 24 pots. Individual pots are represented as points in the figure and are jittered to better visualize individual points with overlapping values. Low diversity treatments are represented in green, high diversity treatments are represented in purple. Straight lines connect means for the same genetic diversity treatment across control and nutrient addition treatments. ns indicates *p* > 0.05, one asterisk indicates *p* < 0.05, two asterisks indicate *p* < 0.01.

*Spartina* plant responses were affected by nutrient addition, but not intraspecific plant diversity. Nutrient addition did not affect aboveground biomass or tiller production (Table 1), but decreased belowground biomass (*F*_1,74_ = 12.76, *p* = 0.00063), and increased percent nitrogen content of leaf tissue (*F*_1,74_ = 119.76, *p* = 5.44 × 10^−10^). Intraspecific genetic diversity levels did not affect aboveground biomass, belowground biomass, tiller production or percent nitrogen content of leaf tissue (Table 1). Mean aboveground biomass of plants in the low and high diversity treatment pots did not differ under control conditions, but aboveground biomass was greater in the high diversity relative to the low diversity treatment with nutrient addition (Figure 2A), though this difference was not significant. While tiller production did not vary with genetic diversity or nutrient addition individually, their interaction did affect tiller production (*F*_1,74_ = 4.4, *p* = 0.04). Tiller production did not differ between low and high diversity treatments under control conditions, but was greater for the low diversity relative to the high diversity treatment with nutrient addition (Figure 2C). Plants produced an average of 2.8 tillers each (range 0–15 tillers) over the 2 months of the study. Mean belowground biomass of plants decreased with nutrient addition, but was not affected by intraspecific genetic diversity (Figure 2B). Mean percent nitrogen content was greater in nutrient addition pots than in control pots, regardless of intraspecific genetic diversity (Figure 2D). The covariate, initial stem height, affected aboveground (*F*_1,74_ = 181.48, *p* < 0.0001) and belowground (*F*_1,74_ = 132.98, *p* < 0.0001) plant biomass, as well as percent nitrogen content of leaf tissue (*F*_1,74_ = 47.12, *p* < 0.0001), but not tiller production (Table 1).

**TABLE 1:** Two factorial MANCOVA univariate tests on mean *Spartina alterniflora* responses by pot (*n* = 79) to nutrient addition and intraspecific genetic diversity treatments. The mean initial stem height, recorded before treatments were applied, is included in the model as a covariate.

	df	MS	*F*	*p*
Aboveground biomass (log transformed)				
Nutrient addition	1	0.049	0.60	0.44
Intraspecific genetic diversity	1	0.12	1.43	0.24
Interaction	1	0.11	1.34	0.25
**Covariate: initial mean stem height**	**1**	**14.83**	**181.48**	**< 0.0001**
Residuals	74	0.085		
Belowground biomass (log transformed)				
**Nutrient addition**	**1**	**2.04**	**12.76**	**0.00063**
Intraspecific genetic diversity	1	0.24	1.47	0.23
Interaction	1	0.13	0.81	0.37
**Covariate: initial mean stem height**	**1**	**21.25**	**132.98**	**< 0.0001**
Residuals	74	0.16		
Tiller production (log transformed)				
Nutrient addition	1	0.00030	0.0013	0.97
Intraspecific genetic diversity	1	0.0023	0.0098	0.92
**Interaction**	**1**	**1.03**	**4.4**	**0.04**
Covariate: initial mean stem height	1	0.24	1.03	0.31
Residuals	74	0.23		
Leaf tissue nitrogen content (%)				
**Nutrient addition**	**1**	**10.18**	**119.76**	**< 0.0001**
Intraspecific genetic diversity	1	0.016	0.18	0.67
Interaction	1	0.03	0.36	0.55
**Covariate: initial mean stem height**	**1**	**4.0**	**47.12**	**< 0.0001**
Residuals	74	0.085		

*Note:* Treatment effects are bolded when the null hypothesis is rejected.

### 3.3 Multilocus Lineage (Clonal) Response Patterns

**FIGURE 3:**
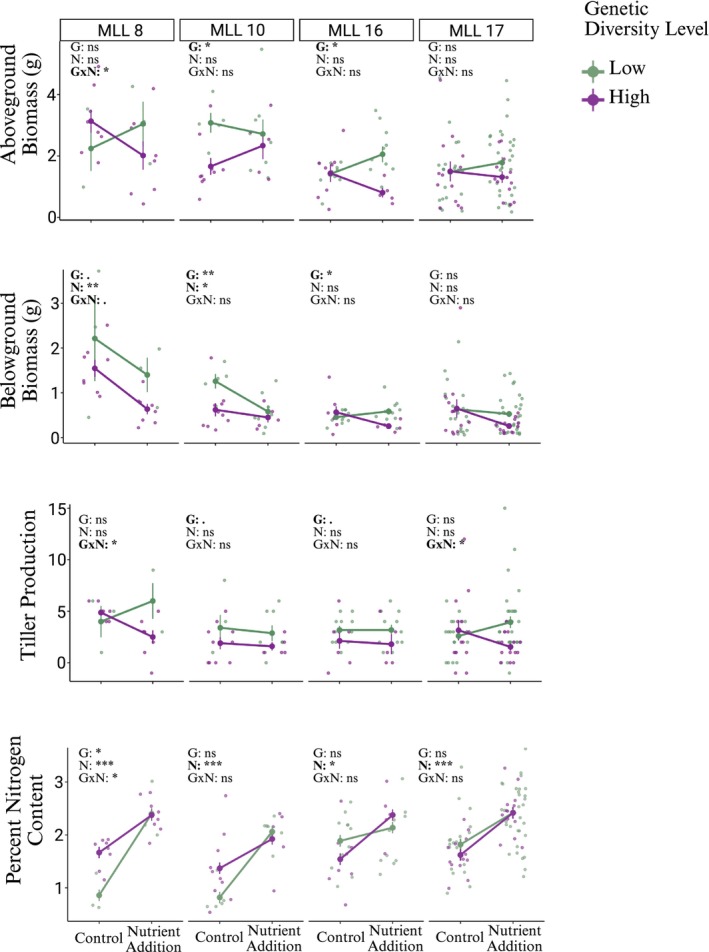
Mean *Spartina alterniflora* responses for the four most abundant multilocus lineages (MLL; clones): MLL‐8 (*n* = 22 pots), MLL‐10 (*n* = 28 pots), MLL16 (*n* = 35 pots), and MLL‐18 (*n* = 78 pots). Shown are mean (±SE) responses for aboveground biomass in grams (top row), belowground biomass in grams (second row), tiller production in number of stems (third row), and percent nitrogen content (fourth row) to the factorial treatment effects of nutrient addition (N), intraspecific genetic diversity (G), and interaction (G×N). Low diversity treatments are represented in green, high diversity treatments are represented in purple. Straight lines connect means for the same genetic diversity treatment across control and nutrient addition treatments. ns indicates *p* > 0.05, one asterisk indicates *p* < 0.05, two asterisks indicate *p* < 0.01.

When we explored response patterns of the four most abundant MLLs to nutrient addition and intraspecific genetic variation, we observed variation in responses among MLLs for aboveground biomass, belowground biomass, and tiller production but similar responses in plant percent nitrogen (Figure 3). While nutrient addition did not affect aboveground biomass for any of the four MLLs, the impacts of intraspecific genetic diversity varied by MLL; MLL‐10 and MLL‐16 both had greater aboveground biomass in low diversity treatments, for MLL‐17 there was no effect of diversity treatments on aboveground biomass, and for MLL‐8 there was an interaction with nutrient addition where aboveground biomass increased in low genetic diversity treatments with nutrient addition (Figure 3 top row, Table S3). These four MLLs demonstrated divergent responses to treatments for belowground biomass as well; nutrient addition decreased belowground biomass for MLL‐8 and MLL‐10, but did not affect MLL‐16 and MLL‐17 (Figure 3, Table S4). Belowground biomass was lower in high intraspecific genetic diversity treatments for MLL‐10 and MLL‐16, had a marginal effect on MLL‐8, and no effect on MLL‐17 (Figure 3 second row, Table S4). Tiller production was marginally greater in low genetic diversity treatments for MLL‐10 and MLL‐16, but we found an interaction between nutrient addition and genetic diversity for MLL‐8 and MLL‐17 (Figure 3 third row, Table S5). Percent nitrogen content increased with nutrient addition for all four MLLs (Figure 3 fourth row, Table S6). Additionally, there was an effect of genetic diversity and the interaction between genetic diversity and nutrient addition for MLL‐8 because plant percent nitrogen was initially greater in high genetic diversity treatments, but became similar to the low genetic diversity treatment with nutrient addition (Figure 3, Table S6).

## 4 Discussion

We found that nutrient addition, but not intraspecific genetic variation, had a strong effect on combined *Spartina* plant responses (aboveground biomass, belowground biomass, tiller production, and percent nitrogen content) over the course of a growing season. Notably, responses to nutrient addition were driven by effects on belowground biomass and leaf tissue nitrogen content, not aboveground biomass or tiller production. The lack of an effect of nutrient addition on aboveground biomass differs from numerous field and mesocosm experiments of similar duration as this experiment that found positive effects of fertilization on *Spartina* aboveground biomass (Denno et al. 2002; Bertness et al. 2008; Sala et al. 2008; Wimp et al. 2010). We may not have observed a positive effect of nutrients on aboveground biomass because this particular *Spartina* population at the Great Bay marsh may invest surplus nitrogen in leaf nitrogen content instead of biomass, as suggested by Murphy et al. (2012) for this site. Our finding that *Spartina* belowground biomass decreases under high nutrient conditions is consistent with previous research (Deegan et al. 2012; Johnson et al. 2016; Hanley et al. 2021). The decreased investment of *Spartina* in roots due to nutrient addition has been shown to make *Spartina* stems more likely to break, leading to detrimental ecological outcomes like salt marsh dieback (Deegan et al. 2012). Similarly, our finding that nutrient addition increases leaf tissue nitrogen content is consistent with previous work (Denno et al. 2002; Murphy et al. 2012). At the high nutrient input amount (55 g N/m^2^), percent nitrogen of leaf tissue was 1.5 times greater in fertilized pots. While we observed some variation in response patterns for the four most common MLLs, overall percent nitrogen consistently increased with nutrient addition. Many studies, representing a variety of focal species, have reported a positive relationship between nutrient additions and plant nitrogen content (Robinson 1994). Further, host plant leaf nitrogen content has been linked to insect herbivore abundance and fecundity (Slansky and Feeny 1977). In salt marshes, *Spartina* percent nitrogen is strongly correlated with stem‐borer larval densities, leading to runaway herbivory and salt marsh dieback (Tomasula et al. 2023). We had presumed that intraspecific genetic diversity would impact plant responses, but we found no evidence for this effect; these *Spartina* responses are instead shaped by environmental factors.

When we examined the impact of nutrient addition and genetic diversity on individual plant responses, we found an interaction between genetic diversity and nutrient addition on tiller production. For the four most common MLLs, we found genetic diversity interacted with nutrient addition to affect tiller production for MLL‐8 and MLL‐17, whereas MLL‐10 and MLL‐16 tended to produce more tillers under low intraspecific genetic diversity conditions, regardless of nutrient addition. Importantly, our results do not support our initial prediction that *Spartina* would use nitrogen to produce more tillers. Tillers, via rhizomes, are the vegetative offspring (ramets) of distinct genets, a growth form which could provide advantages through clonal integration across highly variable local conditions (Bertness 1991; Barrett 2015). Tillering also reduces genetic diversity compared to strict sexual reproduction and produces a patchwork spatial pattern of genets and ramets within populations (Walker et al. 2021; Tomasula 2023). Identifying mechanisms contributing to this wide variation in tiller production would help elucidate the causes and consequences of spatial patterns of ramets and genets in *Spartina*.

Holistically, our results show that intraspecific genetic diversity did not act as a surrogate for interspecific diversity in critical salt marsh ecosystem traits. While nutrient addition interacted with genetic diversity to affect tiller production, genetic diversity alone did not affect any plant responses (aboveground biomass, belowground biomass, tiller production, or percent nitrogen content). The overall pattern was driven by the highly variable responses of different MLLs to intraspecific genetic diversity treatments. Notably, when intraspecific genetic diversity was associated with plant responses, it was in the opposite direction of our prediction; aboveground and belowground biomass increased in low genetic diversity pots for some MLLs. Additionally, while nutrient addition interacted with genetic diversity to affect tiller production, it negatively impacted belowground biomass, increased leaf tissue nitrogen content, and had no impact on aboveground biomass. Our nutrient addition treatment reflected the upper limit of what salt marshes typically experience due to eutrophication (Tomasula et al. 2023; Johnson et al. 2016; Wimp et al. 2019), yet high genetic diversity did not ameliorate plant responses to nutrient addition. This finding contrasts with studies of eelgrass ( *Zostera marina* ) where intraspecific genetic diversity had no effect on shoot density under ambient conditions, but only after extreme stress (e.g., goose overgrazing, heat wave), did genetic diversity influence shoot density (Hughes and Stachowicz 2004; Reusch et al. 2005). Additionally, our findings contrast with two meta‐analyses of intraspecific diversity which have shown that plant genetic diversity, even under ambient conditions, has the strongest positive effect on primary productivity and plant phenotypes (Bailey et al. 2009; Raffard et al. 2018). Previous studies may have employed greater intraspecific diversity by setting up experimental polycultures with plants sourced from diverged populations. Because actual genetic differences have seldom been quantified with genetic markers, the possible impacts in past studies of different degrees of genetic polymorphism are difficult to evaluate.

Since this experiment reflected natural levels of genetic diversity within one population, we likely sampled narrower, but more realistic, levels of genetic variation than some prior studies. Importantly, we used microsatellite genetic distance to identify ramets and genets and quantify levels of genetic polymorphism. This approach accounts for both the clonal reproduction of *Spartina* and the continuous, hierarchical genetic dissimilarity found among individuals in natural populations. The majority of community and ecosystem genetics studies have used genotype richness (here number of MLLs) (Hughes et al. 2008; Bailey et al. 2009), as a metric which implicitly assumes that each vegetative individual (genotype, MLL) used in an experiment is equally dissimilar, or reflects a star phylogeny. However, genotypic richness and genetic dissimilarity are not equivalent; we had pots with only two MLLs that had genetic distance similar to pots with five MLLs. When Abbott et al. (2017) explicitly compared the effect of genotypic richness and genetic relatedness (genetic distance is negatively correlated with relatedness) on eelgrass biomass, they found a strong positive effect of genotypic richness but a negative effect of genetic relatedness, because genotypic richness corresponded to trait variation while relatedness did not. Ultimately, a key underlying assumption of intraspecific genetic diversity studies is that genetic diversity is a proxy for trait variation among individuals, but few studies have been able to clearly link the two (Barker et al. 2019). Abbott et al. (2018) found that genetic relatedness using neutral genetic markers did not correlate with trait variation at the individual level, but it did at the subpopulation level. Yet, that study examined eelgrass under ambient conditions. In our study, neutral genetic markers were useful to compare how different low and high diversity assemblages responded to variable environmental conditions.

While increasing genetic variation at the plot level did not affect trait variation in *Spartina* in this study, it is important to consider the roles of spatial scale and plant reproductive system. *Spartina* is a clonal plant, and genetic variation on a local scale is influenced by the genetically identical individuals (ramets) often clustered together. However, genetic variation may be greater at larger spatial scales. We documented variation in responses to nitrogen addition, in terms of above and belowground biomass, and tiller production, among MLLs in the sampled *Spartina* plants. Because *Spartina* is responsible for the structure of salt marshes, increasing genetic diversity at a larger spatial scale may ensure some MLLs can remain productive, trap sediment, and store belowground carbon even under high nutrient input conditions.

Under the current trajectory of rapid and intensifying environmental change, wild populations are losing genetic diversity (Exposito‐Alonso et al. 2022). Many socially important ecosystems are dominated by relatively few species, and studies of these systems have connected intraspecific genetic diversity to ecological resilience to environmental stressors and to broader community and ecosystem level properties (Hughes et al. 2008; Raffard et al. 2018). In coastal salt marshes, the integrity of the *Spartina* population has consequences for continued societal benefits like storm mitigation in coastal communities, fisheries, and recreation (Gedan et al. 2009; Rippel et al. 2021). We found that nutrient addition leads to lower belowground biomass, which makes *Spartina* populations more vulnerable to loss and dieback (Deegan et al. 2012; Tomasula et al. 2023). Additionally, different MLLs had varying responses to nutrient addition and intraspecific genetic diversity treatments. Thus, while salt marsh restoration should consider the clonal nature of *Spartina* at a local scale, incorporating genetic diversity at a larger scale may bolster biomass trait diversity and differential responses to disturbances such as nutrient addition. Studies such as this one that examine the genetic basis of foundation plant species responses to environmental change will enhance our understanding of ecological resilience.

## Author Contributions

**Jewel Tomasula:** conceptualization (lead), data curation (lead), formal analysis (lead), funding acquisition (supporting), investigation (lead), methodology (lead), project administration (lead), resources (supporting), writing – original draft (lead). **Billie Maguire:** formal analysis (supporting), investigation (equal), visualization (lead), writing – review and editing (equal). **Tyler M. Rippel:** conceptualization (supporting), formal analysis (supporting), investigation (supporting), writing – review and editing (supporting). **Shannon M. Murphy:** formal analysis (supporting), resources (equal), writing – review and editing (equal). **Matthew B. Hamilton:** conceptualization (supporting), formal analysis (equal), funding acquisition (supporting), investigation (supporting), methodology (equal), resources (lead), software (lead), supervision (equal), validation (lead), writing – review and editing (equal). **Gina M. Wimp:** conceptualization (equal), formal analysis (equal), funding acquisition (lead), investigation (equal), methodology (equal), project administration (equal), resources (lead), supervision (lead), writing – original draft (supporting), writing – review and editing (equal).

## Conflicts of Interest

The authors declare no conflicts of interest.

## Supporting information


**Appendix S1:** ece372908‐sup‐0001‐AppendixS1.docx.

## Data Availability

Our data are available for review on Zenodo at this link: https://doi.org/10.5281/zenodo.15178013.
