# Supplementary material for: Nutrient Addition Has a Stronger Effect Than Intraspecific Genetic Diversity on Critical Ecological Responses in a Salt Marsh Foundation Species
Source: Ecol Evol. 2026 Jan 12;16(1):e72908. doi: 10.1002/ece3.72908 (PMC12793896; doi:10.1002/ece3.72908)

**Appendix**

**Table S1.** Test of the probability of finding the observed numbers of genotypes under the null model of sexual random mating by randomizing alleles over individuals within sample locations.

Population Observed Expected

Total 63 394.407 0.001

A 13 79.995 0.001

B 10 78.000 0.001

C 7 78.509 0.001

D 22 77.996 0.001

E 14 79.930 0.001

**Table S2**.Pairwise genetic differentiation for MLLs only in all pairs of sampling locations estimated by *F_ST_* (above the diagonal) under an infinite alleles model and *R_ST_* (below the diagonal) under a stepwise mutation model. Bold indicates a value greater than zero (*p* < 0.05) based on a null distribution generated by random permutation of genotypes among populations.

               A           B           C           D           E

A           --           **0.036**     **0.034**     **0.021**     **0.037**

B           **0.054**     --           0.022     **0.036**     0.021

C           **0.049**     0.030     --           **0.034**     0.021

D           **0.031**     **0.054**     **0.052**     --           **0.028**

E            **0.054**     0.030     0.032     **0.033**     –

**Table S3.** Effects of nutrient input, genetic diversity, and the interaction between nutrient input and genetic diversity on aboveground biomass for the four most common MLLs. Initial plant height was included as a covariate in our factorial ANOVA. Sample sizes were 22 for MLL 8, 28 for MLL 10, 35 from MLL 16, and 78 for MLL 17. Significant results are bolded.

|  | MLL 8 | | | MLL 10 | | | MLL 16 | | | MLL 17 | | |
| --- | --- | --- | --- | --- | --- | --- | --- | --- | --- | --- | --- | --- |
|  | df | F | P | df | F | P | df | F | P | df | F | P |
| Nutrient addition | 1,17 | 1.782 | 0.20 | 1,23 | 1.538 | 0.23 | 1,30 | 1.407 | 0.24 | 1,73 | 0.753 | 0.39 |
| Intraspecific Genetic diversity | 1,17 | 0.019 | 0.89 | 1,23 | 6.29 | **0.0196** | 1,30 | 6.087 | **0.0195** | 1,73 | 1.634 | 0.21 |
| Covariate: initial mean stem height | 1,17 | 5.838 | **0.0272** | 1,23 | 7.942 | **0.0098** | 1,30 | 16.846 | **0.0003** | 1,73 | 51.148 | **5.44E-10** |
| Interaction | 1,17 | 4.758 | **0.0435** | 1,23 | 0.438 | 0.51 | 1,30 | 1.242 | 0.27 | 1,73 | 0.617 | 0.44 |

**Table S4**. Effects of nutrient input, genetic diversity, and the interaction between nutrient input and genetic diversity on belowground biomass for the four most common MLLs. Initial plant height was included as a covariate in our factorial ANOVA. Significant results are bolded.

|  | MLL 8 | | | MLL 10 | | | MLL 16 | | | MLL 17 | | |
| --- | --- | --- | --- | --- | --- | --- | --- | --- | --- | --- | --- | --- |
|  | df | F | P | df | F | P | df | F | P | df | F | P |
| Nutrient addition | 1,17 | 13.213 | **0.0021** | 1,23 | 5.236 | **0.0316** | 1,30 | 1.407 | 0.24 | 1,73 | 0.753 | 0.39 |
| Intraspecific Genetic diversity | 1,17 | 3.718 | 0.07 | 1,23 | 8.117 | **0.0091** | 1,30 | 6.087 | **0.0195** | 1,73 | 1.634 | 0.21 |
| Covariate: initial mean stem height | 1,17 | 7.529 | **0.0138** | 1,23 | 10.53 | **0.0036** | 1,30 | 16.846 | **0.0003** | 1,73 | 51.148 | **5.44E-10** |
| Interaction | 1,17 | 3.413 | 0.08 | 1,23 | 0.948 | 0.34 | 1,30 | 1.242 | 0.27 | 1,73 | 0.617 | 0.44 |

**Table S5**. Effects of nutrient input, genetic diversity, and the interaction between nutrient input and genetic diversity on tiller production for the four most common MLLs. Significant results are bolded.

|  | MLL 8 | | | MLL 10 | | | MLL 16 | | | MLL 17 | | |
| --- | --- | --- | --- | --- | --- | --- | --- | --- | --- | --- | --- | --- |
|  | df | F | P | df | F | P | df | F | P | df | F | P |
| Nutrient addition | 1,18 | 2.271 | 0.15 | 1,24 | 0 | 0.98 | 1,31 | 0 | 0.98 | 1,74 | 0.474 | 0.49 |
| Intraspecific Genetic diversity | 1,18 | 2.223 | 0.15 | 1,24 | 3.036 | 0.09 | 1,31 | 3.612 | 0.07 | 1,74 | 2.542 | 0.12 |
| Interaction | 1,18 | 6.174 | **0.0230** | 1,24 | 0.02 | 0.89 | 1,31 | 0.065 | 0.80 | 1,74 | 5.086 | **0.0271** |

**Table S6**. Effects of nutrient input, genetic diversity, and the interaction between nutrient input and genetic diversity on plant tissue percent nitrogen for the four most common MLLs. Initial plant height was included as a covariate in our factorial ANOVA. Significant results are bolded.

|  | MLL 8 | | | MLL 10 | | | MLL 16 | | | MLL 17 | | |
| --- | --- | --- | --- | --- | --- | --- | --- | --- | --- | --- | --- | --- |
|  | df | F | P | df | F | P | df | F | P | df | F | P |
| Nutrient addition | 1,17 | 38.597 | **9.46E-06** | 1,23 | 22.729 | **8.31E-05** | 1,30 | 4.56 | **0.041** | 1,73 | 34.739 | **1.08E-07** |
| Intraspecific Genetic diversity | 1,17 | 5.355 | **0.0334** | 1,23 | 1.503 | 0.23 | 1,30 | 0.184 | 0.67 | 1,73 | 0.586 | 0.45 |
| Covariate: initial mean stem height | 1,17 | 0.176 | 0.68 | 1,23 | 7.548 | **0.0115** | 1,30 | 0.626 | 0.44 | 1,73 | 17.093 | **9.39E-05** |
| Interaction | 1,17 | 6.349 | **0.022** | 1,23 | 1.438 | 0.24 | 1,30 | 1.023 | 0.32 | 1,73 | 0.277 | 0.6 |

**Figure S1.**  Histogram of pairwise Bruvo’s genetic distances employing a stepwise mutation model for the multilocus lineages (MLLs) identified with a threshold genetic distance of 0.189.


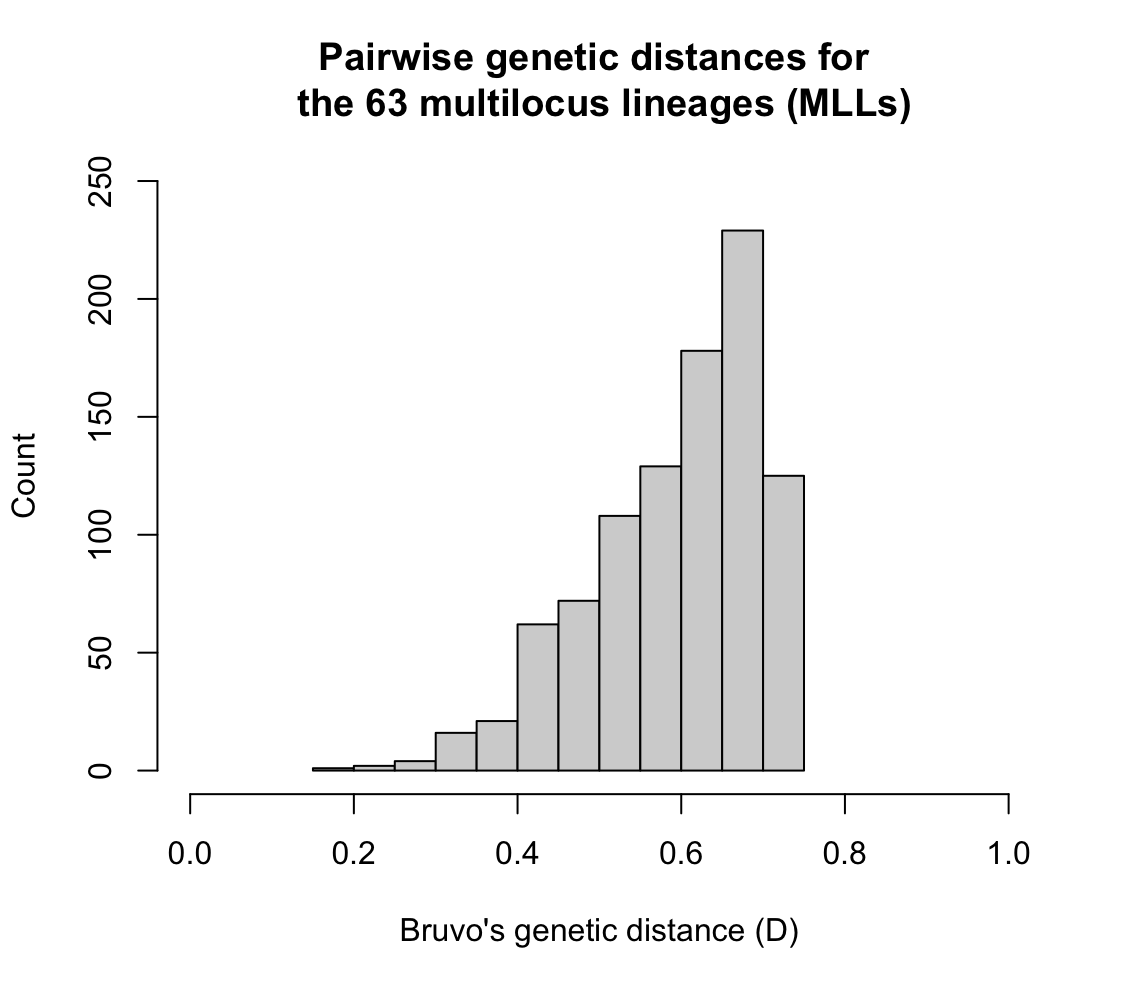


**Figure S2.**  Neighbor joining tree for 63 multilocus lineages (MLLs) based on pairwise Bruvo’s genetic distances estimated from ten microsatellite loci. Branch tip labels give the arbitrary MLL numbers. The table gives counts for the occurrences of each MLL within each marsh sampling location shown in Figure 1.


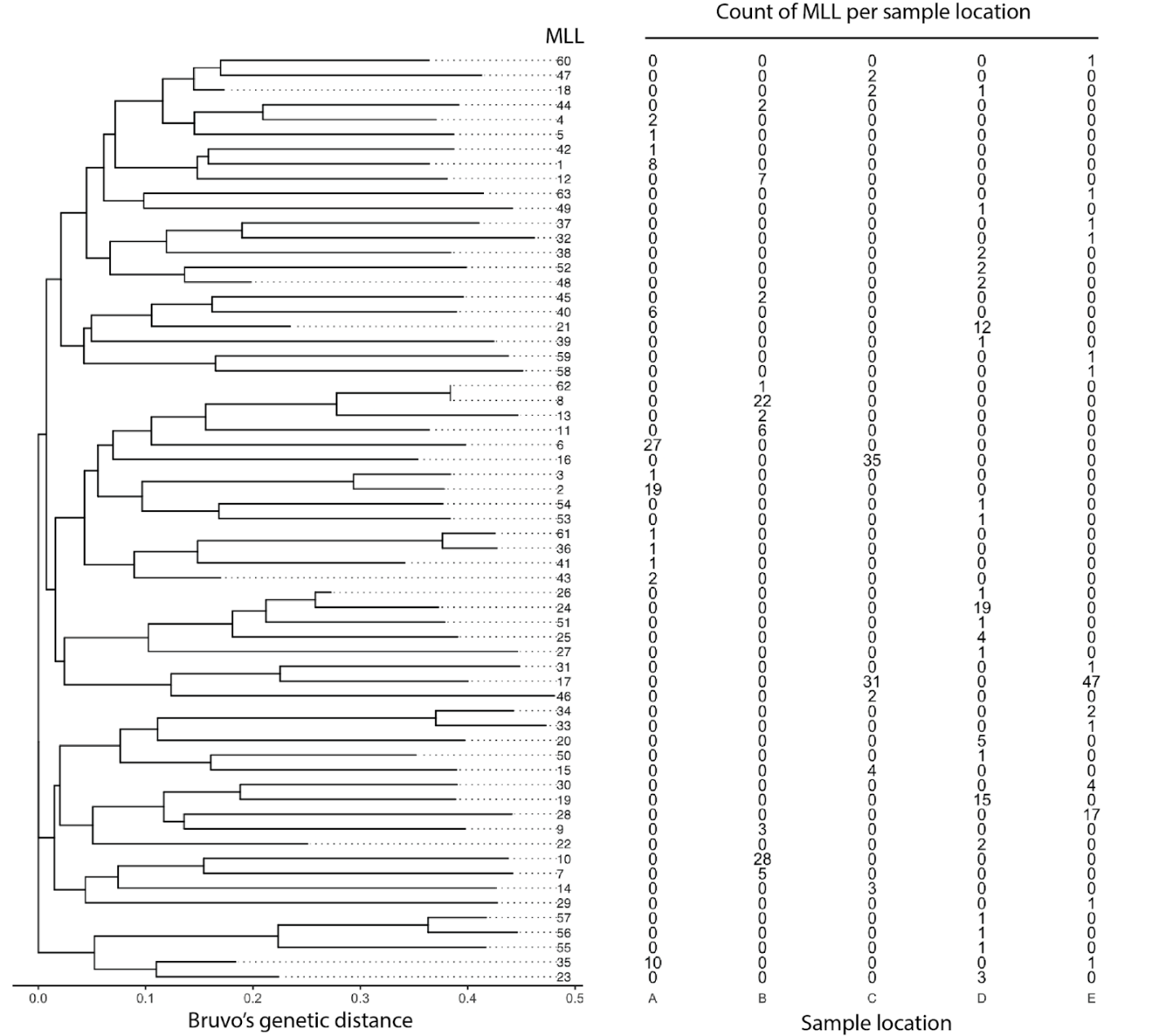

Supplement: Supplementary file 1 — Appendix S1: ece372908‐sup‐0001‐AppendixS1.docx. [file ECE3-16-e72908-s001.docx]
